# Placing engineering in the earthquake response and the survival chain

**DOI:** 10.1038/s41467-024-48624-3

**Published:** 2024-05-20

**Authors:** Luis Ceferino, Yvonne Merino, Sebastián Pizarro, Luis Moya, Baturalp Ozturk

**Affiliations:** 1 University of California, Berkeley, Berkeley, California USA; 2 New York University, Brooklyn, New York USA; 3 Pontificia Universidad Católica de Chile, Santiago, Chile; 4 Servicio de Atención Médica de Urgencia, Santiago, Chile; 5 Universidad Finis Terrae, Santiago, Chile; 6 Pontificia Universidad Católica del Perú, Lima, Perú

**Keywords:** Natural hazards, Epidemiology, Social sciences

## Abstract

Earthquakes injure millions and simultaneously disrupt the infrastructure to protect them. This perspective argues that the current post-disaster investigation paradigm is insufficient to protect communities’ health effectively. We propose the Earthquake Survival Chain as a framework to change the current engineering focus on infrastructure to health. This framework highlights four converging research opportunities to advance understanding of earthquake injuries, search and rescue, patient mobilizations, and medical treatment. We offer an interdisciplinary research agenda in engineering and health sciences, including artificial intelligence and virtual reality, to protect health and life from earthquakes.

## Abstract (web summary)

This Perspective provides insightful discussion in how engineers can aid human health and safety during earthquake disasters. From search and rescue, helping mobilize patients, and securing medical facilities and treatment engineering can work towards bettering earthquake response.

## Introduction

In the second half of the 20th century, earthquake engineers started comprehensive post-disaster field investigations, also known as earthquake reconnaissance, to advance the understanding of infrastructure performance during earthquakes^1^. Notable and impactful post-earthquake field investigations in the United States have triggered upgrades to the standards for seismic design of buildings and retrofit programs. For example, the 1971 San Fernando Earthquake severely damaged many concrete buildings (including hospitals). Field observations following the earthquake led to the 1973 and 1976 Uniform Building Code (UBC)’s new provisions to make concrete buildings more ductile and the Alquist Act’s enactment to build hospitals that remain functional after earthquakes^2–4^. Similarly, field investigations reported unexpected brittle damage in steel moment-resisting frame structures’ beam-to-column connections in the 1994 Northridge Earthquake^5^. These observations led to multiple new provisions in the 1997 UBC, generally considered the “benchmark” building code, to increase the strength and ductility of these connections and to move the code away from using prescriptive provisions towards criteria based on structural performance.

**Fig. 1:**
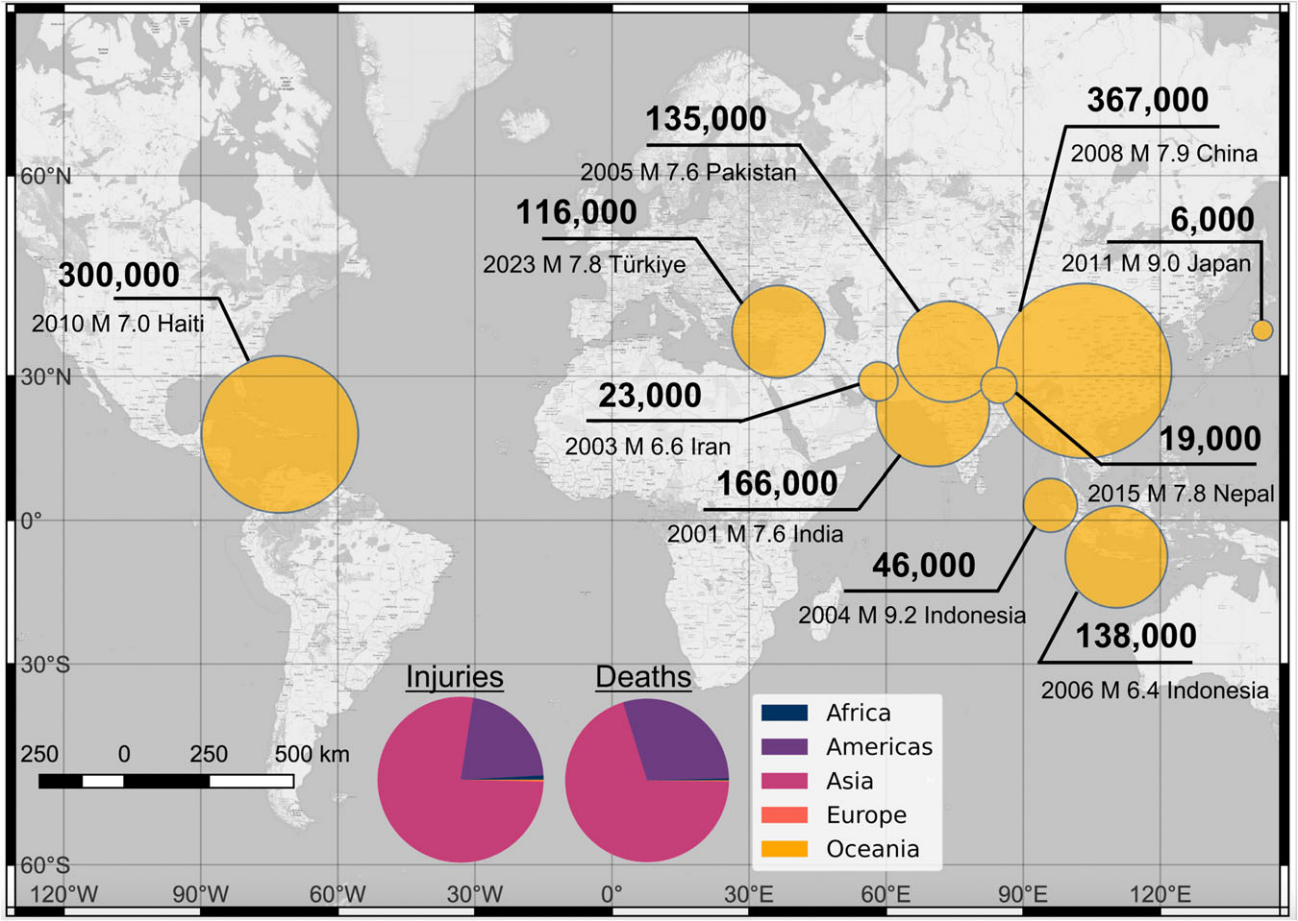
Number of Injuries (in bold text) in the ten deadliest earthquake emergencies in the 21st Century. Notably, 98 earthquakes have injured more than 500 people in each instance since 2000, and 68 earthquakes have killed more than 100 people in each instance since 2000. The size of the yellow circles represents the number of injuries in the emergencies. Pie charts indicate the share of injuries and deaths from all earthquakes across continents.

Post-earthquake fieldwork has tremendously impacted the design and construction of new buildings. This traditional paradigm of learning from earthquakes has the potential to create resilient buildings, relying on the renewal of buildings, i.e., that at some point, old and vulnerable infrastructure will be replaced by new ones designed and built according to appropriate standards. Unfortunately, mass casualty earthquakes and emergency responses over the last decades demonstrate this approach is insufficient to protect people’s lives and health, especially in Asia and the Americas (Fig. 1). For example, in the 21st century, six earthquakes - in India, Pakistan, Indonesia, China, Haiti, and Türkiye - caused more than 100,000 injuries in each instance^6^. The significant casualty events show that despite tremendous learning from field investigations, the focus on infrastructure and the seismic code is still insufficient to protect people.

Retrofit programs have been rare and insufficient in countries of all income levels while infrastructure keeps deteriorating and aging. In addition, rapid urbanization in the Global South has led to the construction of millions of housing units that are seismically vulnerable even though most cities already have modern seismic standards, e.g., in the Caribbean, South America, and Asia^7^. Thus, multiple communities worldwide could face unprecedented emergency responses in future large earthquakes, especially in large cities with high seismic risks^8^.

Post-earthquake field investigations still mainly focus on studying infrastructure’s structural response (e.g., failures) during the ground shaking. In contrast, investigating an emergency also requires studying the response of critical services, especially those that seek to stabilize people’s health in the hours, days, and weeks following the shaking. Earthquakes injure many people suddenly, e.g., more than 100,000 people in the 2023 Türkiye Earthquake^9^. Thus, improving the response to such a massive emergency demands new engineering approaches.

## The earthquake survival chain

**Fig. 2:**
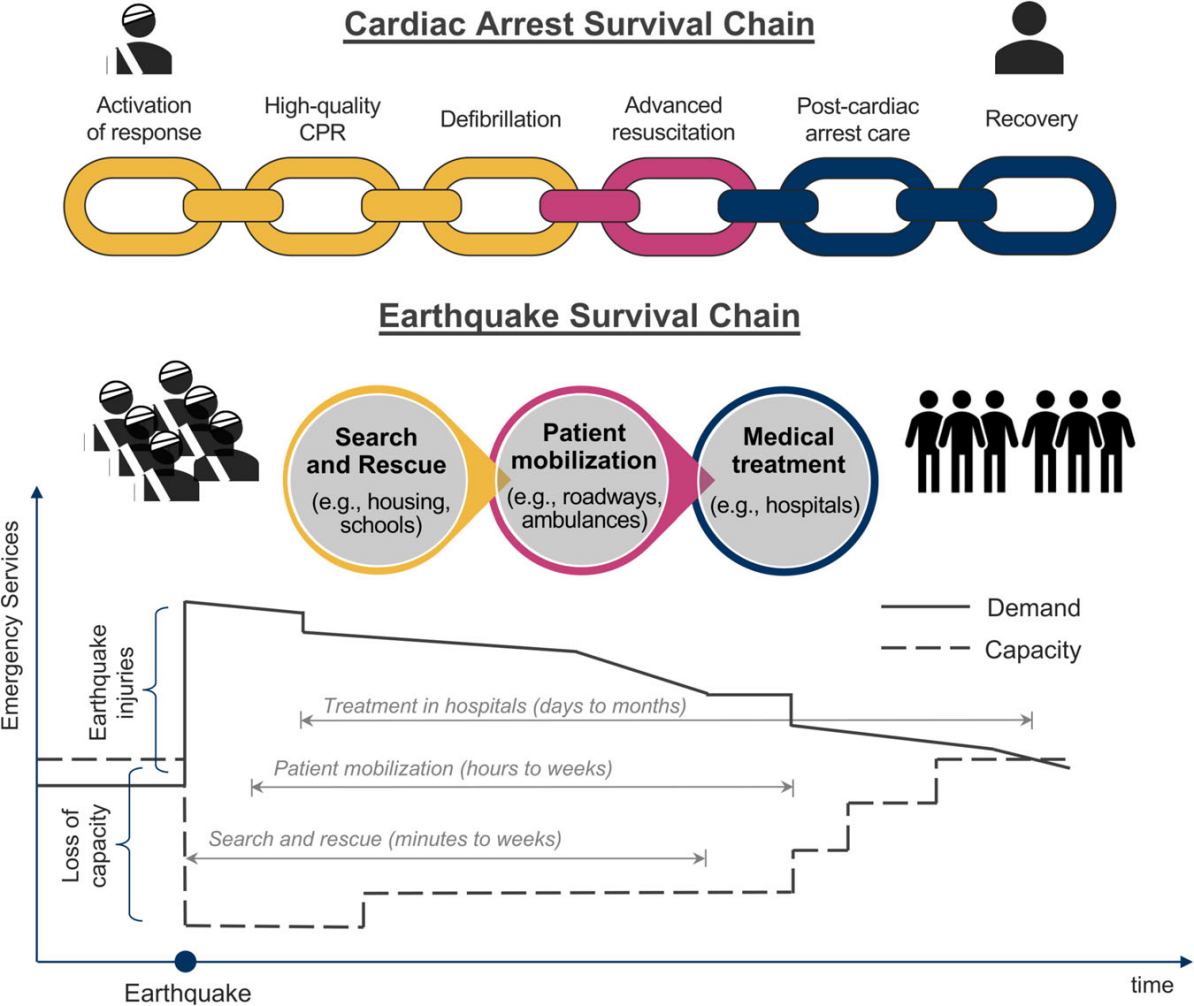
The Earthquake Survival Chain (below) is an analogy to the AHA’s Cardiac-arrest Survival Chain (above). The AHA’s chain and the proposed chain include: first response (yellow), transition (pink), and definite care (blue) stages. The proposed Earthquake Survival Chain includes search and rescue, patient mobilization, and medical treatment. In parenthesis, we include examples of a key infrastructure type (but not the only ones) associated with each link.

To guide the research agenda in disaster emergencies, we review the infrastructure services that become critical after earthquakes. We propose the Earthquake Survival Chain, inspired by the American Heart Association’s (AHA) Chain of Survival^10^, to pinpoint the services that improve the chances of survival and recovery of earthquake victims. AHA’s Chain of Survival focuses on patients undergoing cardiac arrest, the most critical medical emergency since death can occur within minutes. The AHA’s chain uses a systematic approach to characterize five sequential processes (links) for patient survivability (Fig. 2). The AHA’s chain has six links: the activation of the response, high-quality cardiopulmonary resuscitation (CPR), and defibrillation as the first response; advanced resuscitation (and mobilization) as a transition phase; and recovery of patients in hospitals as definite care.

We build on the AHA’s systematic approach to define the Earthquake Survival Chain. Like cardiac arrests, earthquakes trigger sudden emergencies. Many patients require timely life-saving medical services, especially those severely injured. However, earthquake emergencies are larger than a single cardiac arrest as they can cause many injuries at once, and they damage the critical infrastructure needed to respond to the emergency^11^. Thus, the Earthquake Survival Chain’s links become more complex and intertwined (Fig. 2).

The Earthquake Survival Chain becomes active after earthquakes. Patients often have orthopedic trauma injuries, e.g., lacerations, cuts, fractures, and crush syndrome^12,13^. Orthopedic trauma frequently occurs within buildings, especially after large earthquakes. Intense seismic shaking causes heavy infrastructure damage, exposing building occupants to falling hazards, from non-structural elements like heavy furniture to structural elements like concrete slabs^14–16^. Also, earthquakes cause post-earthquake stress-induced conditions, such as ischemic diseases, requiring immediate treatment^17^.

Following the earthquake, the **first link** in the chain is search and rescue as the first response phase (Fig. 2). Earthquake victims can be trapped within heavily damaged buildings, e.g., collapsed housing, schools, and offices (Fig. 2)^18^. Search and rescue activities are needed to locate and extract these victims. Speed is essential as people trapped in the rubble often struggle to survive longer than a few days. Search and rescue teams are frequently under-resourced since areas hit by an earthquake can be vast, and prioritizing the sites for rescue activities becomes critical.

The **second link** is patient mobilization as a transition phase (Fig. 2). Patients need to reach hospitals by their own means or in ambulances^19^. Timeliness is critical, especially for severely injured people, e.g., to prevent excessive bleeding. The transportation system, composed of multiple infrastructure components (e.g., bridges and roads), supports this link (Fig. 2)^20^. Disruptions to transportation infrastructure and lack of mobilization coordination (e.g., not accounting for post-earthquake traffic) can delay treatment delivery for many.

The **third link** is medical treatment and patient recovery as a definite care phase, mainly supported by the hospital infrastructure (Fig. 2). Earthquake patients require various medical resources depending on their specific medical conditions. For example, crush syndrome patients with kidney failure require hemodialysis^21,22^. Patients with limb fractures often require surgery under general anesthesia in an operating room^23,24^. The health outcomes of earthquake victims rely on the hospitals, which is often fragile against earthquakes^13^.

These links for earthquake survivability are strongly connected to specific infrastructure, whose individual performance is crucial in determining the chain’s overall performance. We utilize the earthquake survival chain to identify and propose research opportunities and data needs to improve the understanding of the earthquake survival chain. We highlight how these opportunities can help us prepare and respond better to large earthquakes, starting with how earthquakes injure people and following with the three links.

## Earthquake injuries

To engineer the survival chain, we must first enhance our understanding and modeling techniques that determine the potential future earthquake emergencies. Performance-based earthquake engineering and regional risk modeling provide us with a framework to evaluate future scenarios of building damage and casualties. Accordingly, governments have conducted earthquake casualty studies in North America^25–29^, South America^16,30,31^, Europe^32–34^, and beyond^35^. These studies rely on the canonical casualty risk framework, HAZUS (or similar approaches), established by the Federal Emergency Management Agency (FEMA)^36^. This approach rigorously combines earthquake hazard, exposure, and vulnerability to predict earthquake injuries and fatalities in each building^16,30^. Researchers have successfully applied this framework globally. However, since its inception 30 years ago, tremendous progress in earthquake engineering, epidemiology, and disaster medicine has been made, pointing to gaps in this canonical framework.

**Fig. 3:**
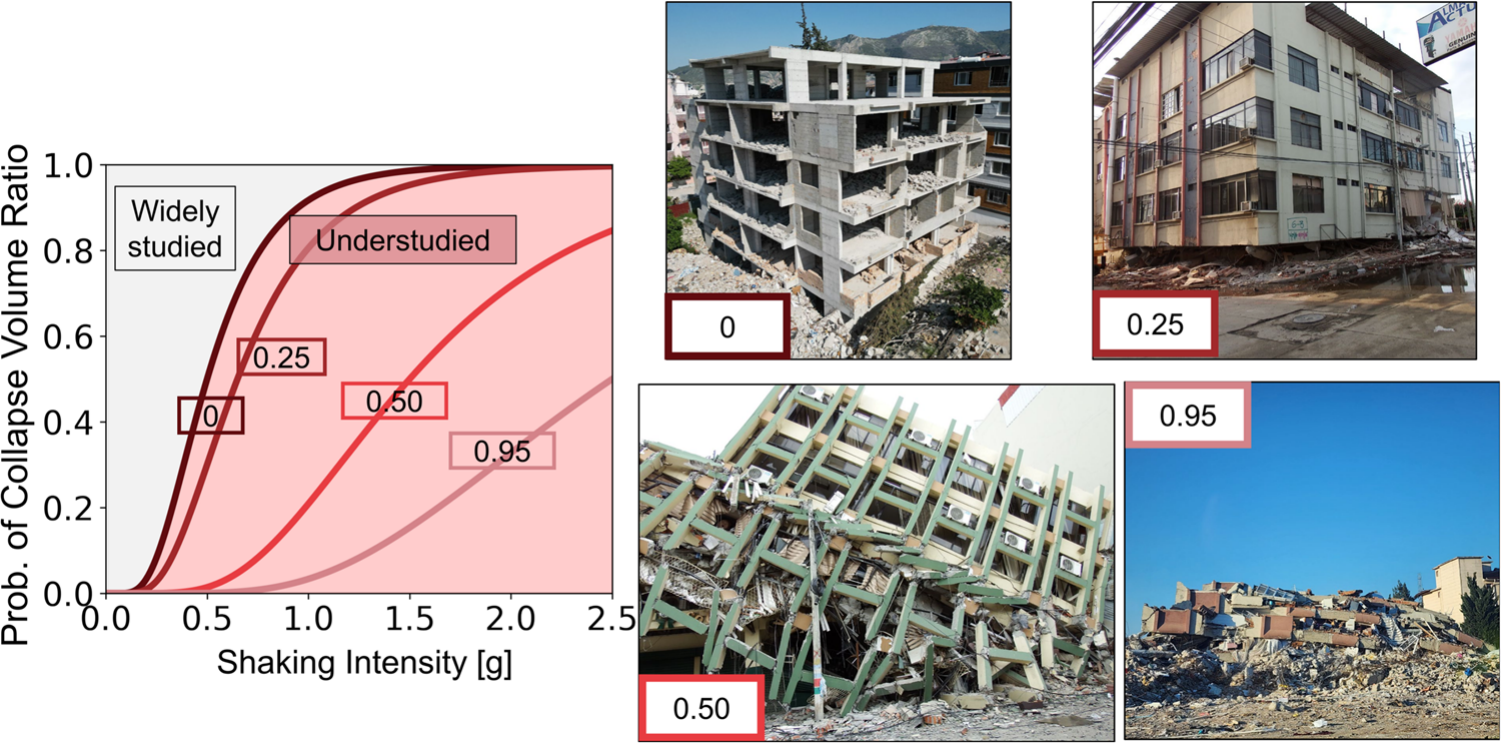
Current models mainly focus on damage levels that do not go beyond the onset of collapse and cannot assess collapse volume ratios, defined as the ratio of lost safe spaces due to local structural collapses within buildings. This drawback limits the ability of engineering models to predict earthquake casualties because structures with higher collapse volume ratios r have more high-severity injuries. Collapse volume ratios (indicated in rectangles) go from small to large from the left to the right.

Casualty models assume a coarse representation of aggregated building damage, missing spatial resolution to reproduce the physical mechanisms that cause injuries. Earthquakes injure people (e.g., with bruises, lacerations, and fractures) due to physical impacts in specific areas rather than the entire building. In contrast, casualty models use uniform likelihoods of casualties throughout entire buildings^16,25,26,30–37^. For example, concrete buildings with extensive damage have a ~1% injury likelihood in all building spaces^36^. However, earthquakes in New Zealand, Iran, and Türkiye have shown that injury occurrence is highly heterogeneous, and people are severely injured (e.g., compound bone fractures) mainly where structural components fail^38,39^. To account for these effects, researchers have defined the “collapse volume ratio” as a measure of the reduced safe volumes within buildings due to partial or total structural collapse^32,40–42^. The collapse volume ratio varies from 0 to 1, where 0 represents a fully standing building with no partial collapses, and 1 represents a full pancake mechanism where all floors collapse (Fig. 3). Partial collapses are in between these values. For example, a four-story building where the first floor collapses due to a soft story mechanism and all the other floors remain standing has a collapse volume ratio of 0.25 (Fig. 3). Researchers have found that the likelihood of deadly injuries is significantly higher in buildings with large collapse volume ratios (Fig. 3)^12,40^.

While significant advancements in understanding and modeling structural responses to earthquakes have been achieved in the last decades^43,44^, these efforts have not focused on studying the structure behavior beyond the onset of collapse, i.e., when collapse volume ratios are bigger than zero. For example, fragility functions, which measure the likelihood of exceeding a damage level for different ground shaking intensities, have been developed for slight, moderate, extensive, and complete damage for many structural archetypes (Fig. 3)^36^. These functions allow us to evaluate key infrastructure risk metrics, such as repair and replacement costs, building downtime, and potential retrofits. However, our understanding and modeling techniques of various damage beyond the onset of collapse, at significant levels of structural deformation, is still limited, preventing current casualty models from having the granularity to assess these local mechanisms triggering high-severity injuries (Fig. 3).

Performance-based engineering framework must build on the concept of (un)safe spaces within buildings to enhance casualty modeling. To do so, we must enhance the granularity of traditional damage assessments of non-structural and structural components to explicitly reproduce the physical mechanisms of trauma injuries within buildings. Exciting opportunities arise from challenging the binary definition of complete structural damage, i.e., when a building is unrepairable, by putting forward a more granular classification based on failure modes leading to higher and more severe injuries. Continuous (rather than binary) variables representing complete damage with different levels of collapse volumes can improve the predictability of earthquake casualties. Datasets capturing various structural collapses are rapidly increasing due to the availability of novel high-resolution satellite imagery and drone close-view footage^45^, which can supplement more limited structural inspections. Historically, building collapse data have been highly perishable as debris removal activities can rapidly demolish heavily damaged buildings before reconnaissance missions can document them. Thus, these new data collection modalities can, for the first time, allow us to study granular structural failures and injuries systematically and comprehensively.

In addition, we can elevate the precision of casualty risk models by creating a more refined categorization of earthquake injuries. Current casualty models have only four injury categories and thus are myopic to the breadth of earthquake patients’ medical diagnoses and needs^16,26,33,36^. For example, patients with crush syndrome or exposed limb bone fractures are considered in the same category (severity three)^36^. While both require immediate treatment, they demand different medical resources and procedures. Crush syndrome patients with kidney failure need hemodialysis^21,46^. In contrast, upper and lower limb fracture patients require a surgical procedure under general anesthesia in an operating room (e.g., internal fixation, debridement, skin grafting)^46,47^. To enhance the resolution of injury categories, earthquake engineering and emergency medicine researchers must work together to develop new survey tools that, unlike existing ones, can help us document earthquake impacts on buildings and people together. Through interdisciplinary earthquake field investigations, we can extend existing epidemiological surveys that can collect injury profile information and link them to local building failures. In addition, medical records after earthquakes can also offer rich information on injury types and medical treatments. Mass-casualty earthquakes, such as the 2023 Türkiye Earthquake, provide an opportunity to collect large and refined injury datasets due to the unfortunately large number of injured people and building collapses. Even if surveys happen after collapsed buildings are cleaned, interdisciplinary teams can collect injury data by interviewing affected people and couple these datasets with drone or satellite imagery documenting granular failures to their buildings, as described earlier.

## Search and rescue

Urban Search and Rescue (USAR) activities can start minutes after the earthquake as part of the first response. USAR teams focus on people within buildings who cannot exit safely by their own means^18^. Frequently, these victims are already injured in heavily damaged buildings. Thus, USAR operations are extremely challenging as they must extract survivors underneath heavy rubble and debris. At the same time, USAR operations seek to minimize the risks to the rescuers as buildings can become unstable, especially during aftershocks^18^.

In large emergencies, USAR teams are significantly under-resourced due to the excessive number of collapsed buildings and victims, e.g., 2023 Türkiye, 2010 Haiti, 2008 China Earthquakes^9,48,49^. Thus, they must make complex decisions about prioritizing their resources to promptly rescue as many people as possible. USAR teams consider two critical factors before entering damaged buildings: the likelihood of the victim’s survival and the time to rescue a victim, and decision-making often unfolds at neighborhood and building levels.

First, USAR teams, in coordination with local emergency agencies, must rapidly inspect entire neighborhoods to identify buildings with trapped survivors. The primary strategy consists of searching buildings with survivable voids, i.e., spaces that remain relatively intact even when the surrounding structure has collapsed, where a person could endure long enough to be rescued^18^. In previous earthquakes, people have survived in those spaces for multiple days, e.g., neonates rescued from a collapsed nursery ten days after the 1985 Mexico Earthquake^50^. USAR teams conduct extensive visual inspections to identify the presence of voids, such as gaps in the rubble, tilted walls, or other irregularities. They also rely on local information and dogs to locate those missing. Current sensing technology can further enhance searching for survivors underneath debris, e.g., thermal cameras, microphones, radar, and radio^51–55^. However, these technologies cannot be extensively and effectively deployed because they demand significant USAR resources (often experienced operators) and still expose rescuers to risks from approaching unstable structures, e.g., to place microphones.

**Fig. 4:**
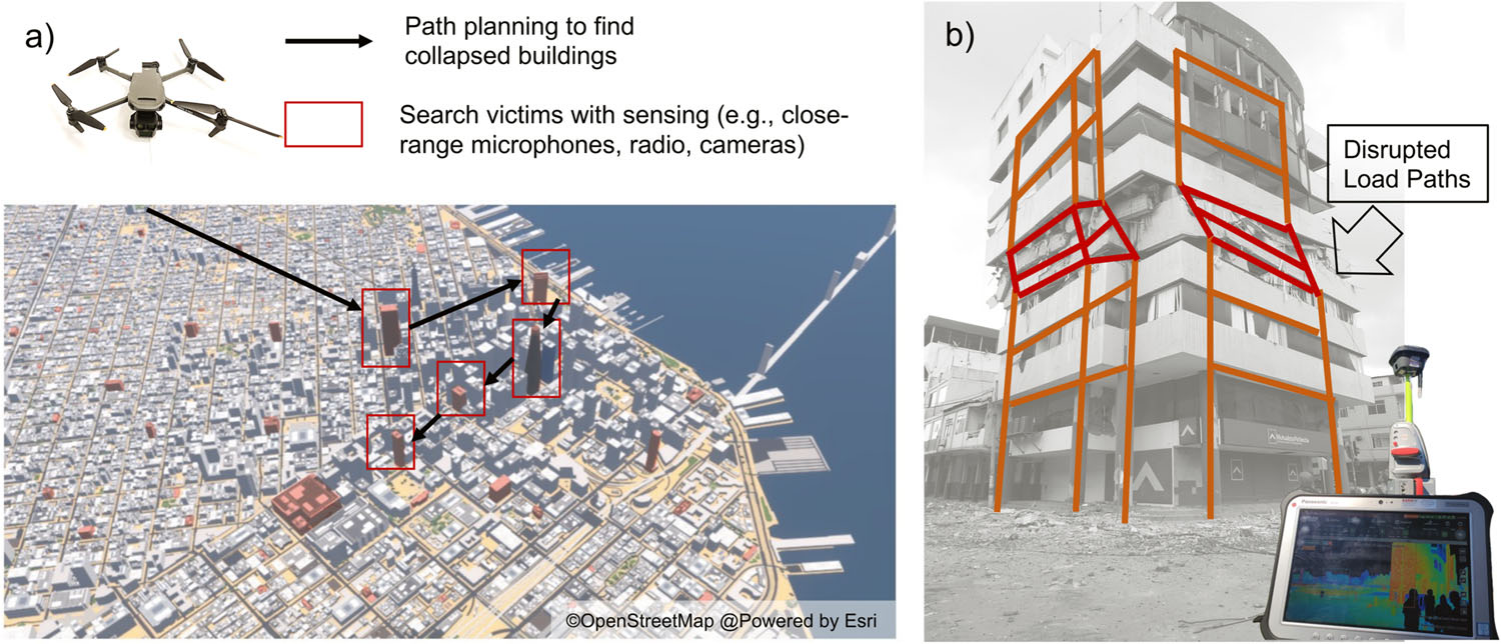
Engineering search and rescue during earthquake emergencies. **a** Engineering-informed simulations to train robots (e.g., drones) to visit the most damaged and dense buildings, searching for trapped people. Map created using Street GL Software available under an © MIT License and Copyright © 2020-2023 StrandedKitty. **b** Mobile lidar to assess damage dynamically through artificial intelligence.

New robotic technologies, like unmanned aerial vehicles (UAV) and ground vehicles (UGV), show a promising avenue to overcome these limitations^56,57^. For example, drones can carry sensors (e.g., microphones, thermal cameras) to increase the coverage of inspections for survival voids, leveraging their high speed on air and enhanced mobility to approach unstable structures without risking rescuers’ lives. Autonomous systems have improved dramatically due to computational power and artificial intelligence (AI) breakthroughs and are currently used for industries like manufacturing^58^. Many autonomous systems are trained to make decisions after learning from extensive datasets of humans’ actions or the robots’ interactions with the environment. For example, warehouse robots are trained to pack, sort, and move products using extensive computer simulations of warehouse activities and inventories^59^. However, robotic technologies still lack improvements to be widely used in post-earthquake search and rescue. Unlike manufacturing, post-earthquake scenes are highly uncertain and dynamic, limiting the autonomy of these systems to move through debris, approach unstable infrastructure, and effectively seek survivable voids. Robots must be trained with extensive data representative of various post-earthquake conditions to release the power of autonomous systems and artificial intelligence (AI) for search and rescue. While gathering datasets from USAR teams in actual search and rescue operations seems unfeasible without compromising their success, autonomous systems can leverage simulated virtual and physical environments representing post-disaster conditions. Examples of physical environments representing disaster conditions already exist where USAR teams train and perform drills, e.g., the Disaster City in Texas, US^60^. Systematically collecting these teams’ decisions is fundamental to improving autonomous systems. Creating similar disaster labs that can reproduce the various conditions (e.g., collapse rates, building densities, survivable void sizes) is critical to making these systems work in future search and rescue. Encouragingly, novel hyper-resolution earthquake risk models can help elucidate future earthquake consequences (e.g., number of injuries, deaths, collapsed buildings) affecting entire cities and create virtual environments to train these systems more extensively (Fig. 4)^16,30,61^. Multiple regional risk models have already been created and used in multiple cities to develop informed policies for risk reduction, considering rich information on earthquake hazards and building vulnerabilities and exposure^7,25^. Combined with virtual environments, these risk simulations can help collect information on the USAR team’s decision-making for many possible damage scenarios. Analyzing and documenting the USAR best strategies to identify buildings with survivable voids in these virtual environments can help create novel and rich datasets to train robots for an enhanced search of earthquake victims.

Second, SAR teams must elaborate and execute plans to enter damaged buildings, making complex decisions that balance victims’ survival odds and teams’ risks^18^. They must establish entry points and rescue paths to reach the voids where victims are trapped. These decisions require structural stability judgments in infrastructure that is partially or fully collapsed, sometimes without structural engineering expertise on the team. They must also determine whether they need to shore unstable parts of the structure, and such decisions have critical implications in rescue times and the use of light or often more scarce heavy equipment. Decisions on debris removal to reach the victims also require structural engineering judgment to avoid compromising the elements that become load paths supporting the collapsed or semi-collapsed infrastructure. USAR teams ponder victims’ survivability time and often accept higher risks to focus their efforts on reaching the victim instead of ensuring the stability of the structure. While making rapid and reliable structural assessments is essential for USAR teams, few field investigations and structural engineering research have focused on the stability of already collapsed infrastructure. Structural engineers mainly focus on characterizing the structure’s behavior up to the onset of collapse to enhance seismic standards and avoid collapse prevention, as mentioned previously. Furthermore, systematic data on buildings where search and rescue activities were conducted is exceptionally scarce, creating a fundamental research gap to create better engineering methods to assess structural stability in damaged infrastructure.

Field investigations need to document these buildings more comprehensively. Post-disaster structural inspections can be linked to USAR teams’ reports to study the entry points and paths, documenting different infrastructure collapse modes (e.g., sideways versus vertical), debris removal equipment (e.g., heavy versus light), shoring techniques, and times to rescue. The systematization of such information has the potential to improve the understanding of stability in collapsed infrastructure to enhance shoring techniques and the estimation of time to rescue, which is vital in helping USAR teams make decisions on rescue strategies. Furthermore, close-range sensing technology for damage identification also has the potential to provide enhanced real-time information regarding the stability of buildings during rescue operations. Due to increased image data of infrastructure affected by earthquakes, new AI (e.g., computer vision) models have been trained to detect damage (e.g., cracks) in different structural components like beams and columns^62,63^. However, little progress has been made in assessing the structure as a whole to identify disruptions to the structures’ load paths for stability, which is crucial for the buildings where USAR teams conduct search and rescue operations. Computer vision models that identify load paths with cameras can help USAR teams (which sometimes do not have a structural engineer in the team) make better decisions on whether damage to structural components affects the stability of the structure and provide critical information on key points for shoring. In addition, the use of LiDAR onsite, in combination with images, can further enhance damage predictions. LiDAR can dynamically detect displacements and deformations, indicating the onset of instabilities in the structure (Fig. 4)^64^. AI models that couple images and LiDAR scans have the potential to improve damage detection to help USAR teams make more informed decisions and reduce risks during their operations.

## Patient Mobilizations

Many people travel through streets, roads, and bridges in an emergency. In mass-casualty earthquakes, thousands of severely injured people must travel to seek timely medical treatment, e.g., 2010 Haiti, 2008 China, 2011 Japan Earthquakes (Fig. 1)^49,65–67^. Earthquakes with fewer casualties can also trigger large-scale patient mobilizations. For example, hospitals were damaged due to the M 8.8 2010 Chile earthquake and had to evacuate and transfer 2,000 to 3,000 patients^68^.

**Fig. 5:**
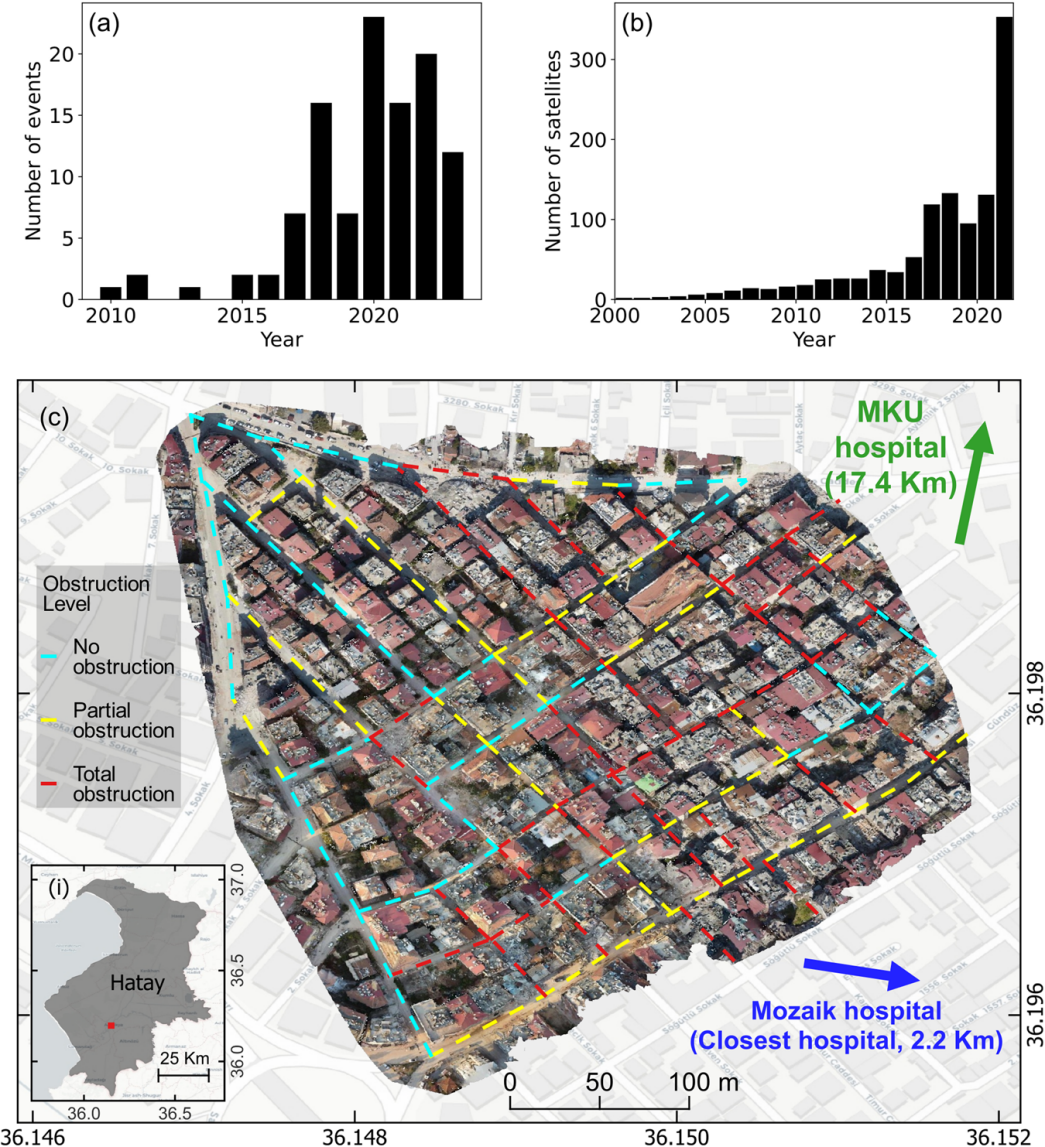
Mapping road disruptions for patient mobilizations after earthquakes with satellite and aerial imagery. **a** The number of events attended by the Maxar’s Open Data Program. **b** The number of earth observation satellites. Source: Union of Concerned Scientists. **c** UAV-based aerial image recorded in Hatay. Dashed lines denote the level of obstruction in the roads/alleys. The blue arrow depicts the direction of the closest hospital, which was not functioning after the earthquake, and the green arrow shows the direction of Mustafa Kemal University (MKU) Hospital, the only working hospital in Hatay after the earthquake. Inset (i) shows the location of the close view in Hatay. Map created using the Free and Open Source QGIS^99^ with open data from OpenAerialMap^100^.

Emergency medical services (EMS), hospitals, USAR teams, and affected communities must mobilize injured people, but mobilization decision-making is challenging because infrastructure failures and post-earthquake traffic conditions can remain largely unknown during emergencies. In cities, post-earthquake mobility is heavily coupled with damage to transportation, residential, and healthcare infrastructure. People can overload the highways that remain functional if many others fail, e.g., due to the collapse of bridges, especially if the transportation system lacks redundancy. Also, collapses of residential buildings can block streets (Fig. 5c)^69,70^. In our deployment to Hatay, USAR teams reported that emergency vehicles could not move through many affected neighborhoods because of the building debris after the 2023 Türkiye earthquake. In addition, many people travel to the same destinations after large earthquakes, rapidly increasing traffic congestion. The 2023 Türkiye earthquake injured 30,000 people in Hatay^9^, and many went to the Mustafa Kemal Hospital, the only hospital (out of 20) that remained functional after the earthquake in the region^71^. As a result, traffic congestion was high on roads nearby, causing further delays for hospitals to receive patients and medical resources.

However, post-earthquake investigations have not been able so far to establish engineering methods to identify road disruptions at a regional scale and to untangle the dependencies between post-earthquake mobility and infrastructure damage. A lack of regional data has mainly been the problem due to its extreme perishability. In an emergency, responders focus on repairing and unblocking roads rather than systematically documenting the damage and mobility. Nevertheless, novel, rich, and large-scale datasets are giving engineers a tremendous opportunity to address these gaps. We point out these new data streams (e.g., satellite imagery, global navigation satellite system (GNSS) data from mobile phones) and outline paths to develop regional assessments of transportation damage and enhance the knowledge of post-disaster mobility.

First, remote sensing, powered by AI, can bestow new methods to identify local road disruptions at large spatial scales. In the last decade, there has been a surge in the deployment of Earth observation satellites (Fig. 5b), and their critical role in identifying damaged housing infrastructure and landslides in large regions after disasters has been widely recognized (Fig. 5a). Satellite imagery can be complemented with unmanned aerial vehicle (UAV) images. While UAVs have lower coverage than satellite imagery, UAVs can carry cameras to capture close views of infrastructure failures with resolutions of a few centimeters.

Researchers can use high-resolution satellite or UAV imagery to predict building damage and landslides through AI, e.g., convolutional neural networks (CNNs) and semantic segmentation^72–76^. Researchers can also use lower-resolution satellite imagery, resulting in coarser predictions but with sizeable geographical coverage^75,76^. However, training AI models for damage detection requires substantial data. Thus, remote sensing researchers have dedicated significant efforts to compiling post-disaster satellite imagery (including but not limited to earthquakes) and annotating building damage and landslides. In contrast, datasets on transportation systems after disasters are at a more nascent stage.

We must create comprehensive datasets for transportation systems, but unlike the research for buildings and landslides, we do not have to start from scratch. A promising approach is to leverage existing datasets that already capture multiple mechanisms of transportation system disruptions. For example, we can leverage the catalogs of building damage and landslides from previous disasters because, in many cases, these failures have resulted in blocked streets and roads (Fig. 5c). Existing datasets on building collapses and landslides are currently prominent and keep growing. For example, until 2019, the xBD satellite imagery dataset had 850,736 building annotations, of which 31,560 were from destroyed buildings after disasters^74^. We can extend these datasets by annotating the failures that lead to street and road blockages. Furthermore, researchers can also capitalize on transfer learning, an AI approach to reuse CNN models trained on one task for its use on a second related task. Researchers can exploit existing AI models’ knowledge of building damage and landslide identification to extend it to disruptions in transportation lines.

Second, other new data streams can help us elucidate post-earthquake mobility and reveal its dependency on infrastructure failures. Post-earthquake mobility is difficult to characterize because it can vary dramatically from earthquake to earthquake and from region to region. However, datasets, such as Call Detail Records (CDRs) and smartphone locations, can provide rich and large-scale information on mobility during disaster emergencies^77–79^. CDRs track service loads on cell phone towers, and while they cannot identify people’s precise location, they capture mobility between areas covered by different towers. In addition, smartphone data, often collected by tech companies (e.g., Google, Facebook, Apple), contains location information with a spatial accuracy of a few meters and observation frequency of a few minutes through the device’s Global Navigation Satellite System (GNSS). These GNSS datasets capture hyper-local mobility patterns during emergencies to enable the analysis of the ties between infrastructure failures and traffic congestion at the level of individual infrastructure assets.

For illustration, consider the scenario in Fig. 5c. The neighborhood’s west side has only partial obstructions, whereas the east side was completely blocked due to the debris from medium-rise concrete buildings. Consequently, mobile phone GNSS data would have enough resolution to capture how people moved towards the west side in search of help, avoiding the disrupted streets. The data would have also captured USAR teams accessing the affected neighborhood through the west side, as reported in our interviews with the Hatay firefighters during our fieldwork. Mobile phone data (and also CDRs at a coarser level) can help us learn these local patterns over large spatial extents, and such information has the potential to better guide the mobilization of patients, as well as other emergency response activities. For example, emergency responders could identify road blockages more rapidly to avoid them or plan on debris removal activities. They could also better guide the traffic by rerouting car flows before jams occur. We envision these mobility datasets will be exploited comprehensively soon to study earthquake emergencies as they become more widely available. With more mobility data, we can characterize the dynamics of origin and destination points and flows during earthquake emergencies, tracking variations from regular (pre-earthquake) mobility flows. We can also couple this information with locations of multiple building and infrastructure uses, e.g., OpenStreetMap, on housing, transportation, and healthcare. Connecting these two datasets holds tremendous potential to untangle the dependencies between post-disaster mobility, infrastructure, and damage.

## Medical treatment

Thousands could seek medical treatment in hospitals after earthquakes. To provide these services, seismic standards aim to have strong enough hospitals to sustain full operations even after large earthquakes. Nevertheless, earthquakes keep disrupting hospitals, often without causing much damage. Many hospitals that experience no (or slight) damage to their buildings’ structural elements (i.e., those from the central load-resisting system) can stop all operations if non-structural building components fail. For example, the Christchurch Hospital had no structural damage after the 2011 New Zealand Earthquake. However, broken water pipes and tanks flooded the upper floors, disrupting critical services like the blood bank^80,81^. In addition, failures of backup generators further disrupted intensive care units, the radiology department, and emergency services. Similar observations were drawn from the 2016 Japan and 2010 Chile earthquakes. In Chile, 85% of the affected facilities reduced their radiology service capacity due to insufficient backup power^82^. In Japan, 80% of the surveyed hospitals had failures in their water connections, which resulted in disruptions to some critical services (e.g., hemodialysis and sterilizations) or even complete evacuations in some cases^83,84^.

Accordingly, engineers have studied the vulnerabilities of non-structural building components. For example, engineers have conducted numerical analyses of non-structural components’ failures through non-linear dynamic analysis that couples the predicted building’s floor acceleration to the motions and deformations of the non-structural elements^85–87^. With this approach, engineers can capture various failure modes, including overturning, sliding, and in-plane and out-of-plane instabilities. In addition, engineers have conducted laboratory tests to assess non-structural components’ vulnerabilities. Notable landmark experiments in the United States^88,89^ and Japan^90–92^ have subjected entire hospital areas (e.g., intensive care units, operating rooms) with multiple non-structural components and medical equipment inside to ground motions on shaking tables. Capitalizing on these studies, researchers have elaborated engineering models to predict failures in building components within hospitals, evaluating how physical damage to building components can disrupt hospital services^86,93,94^.

Despite the tremendous efforts to characterize physical vulnerability, however, post-earthquake observations increasingly suggest that the functionality of hospital services relies heavily on human factors^83^. Immediately following an earthquake, healthcare staff must make complex decisions ranging from keeping full operations to fully evacuating hospitals. Post-earthquake observations indicate that two fundamental human factors are essential: risk perception and service adaptability.

First, the chief medical staff’s perception of risk is critical because they must ensure the safety of patients and the entire medical personnel. Decision-making is easier in cases of extensive structural damage as cracks in structural elements become noticeable. In this case, the chief medical staff has no option but to evacuate hospital buildings fully (or almost fully). However, decision-making becomes harder in buildings with slight structural damage. Risk perception plays a critical role in these cases. For example, after the 2023 Türkiye earthquake, the chief medical staff decided to fully evacuate the Mustafa Kemal University Hospital, the only functional hospital in Hatay, due to safety concerns following an aftershock (two weeks after the mainshock) that caused visually notorious damage. However, the damage did not compromise the building’s structural integrity. It was only a detachment of concrete cover and mortar in the upper story due to pounding on the seismic gap. Similarly, after the 2016 Kumamoto Earthquake, four out of nine hospitals were evacuated mainly due to safety concerns, even though the buildings’ main structures were not compromised^83^.

Second, medical staff shows tremendous adaptability following earthquakes, resulting in the plasticity of multiple healthcare services. Instead of treating hospital service areas rigidly, healthcare staff can reconfigure areas to provide and expand critical services that otherwise would be lost. In previous earthquakes, medical staff have been able to move critical services from damaged or disrupted areas to other building interior or exterior spaces. For example, after the 2011 Earthquake, the Christchurch Hospital moved its triage area to the parking lot^81^. Similarly, after the 2023 Earthquake, the Mustafa Kemal University Hospital’s staff in Türkiye moved their surgery activities from the upper floors to the first story. Following the aftershock two weeks later, the medical staff moved their emergency department to the parking lot.

**Fig. 6:**
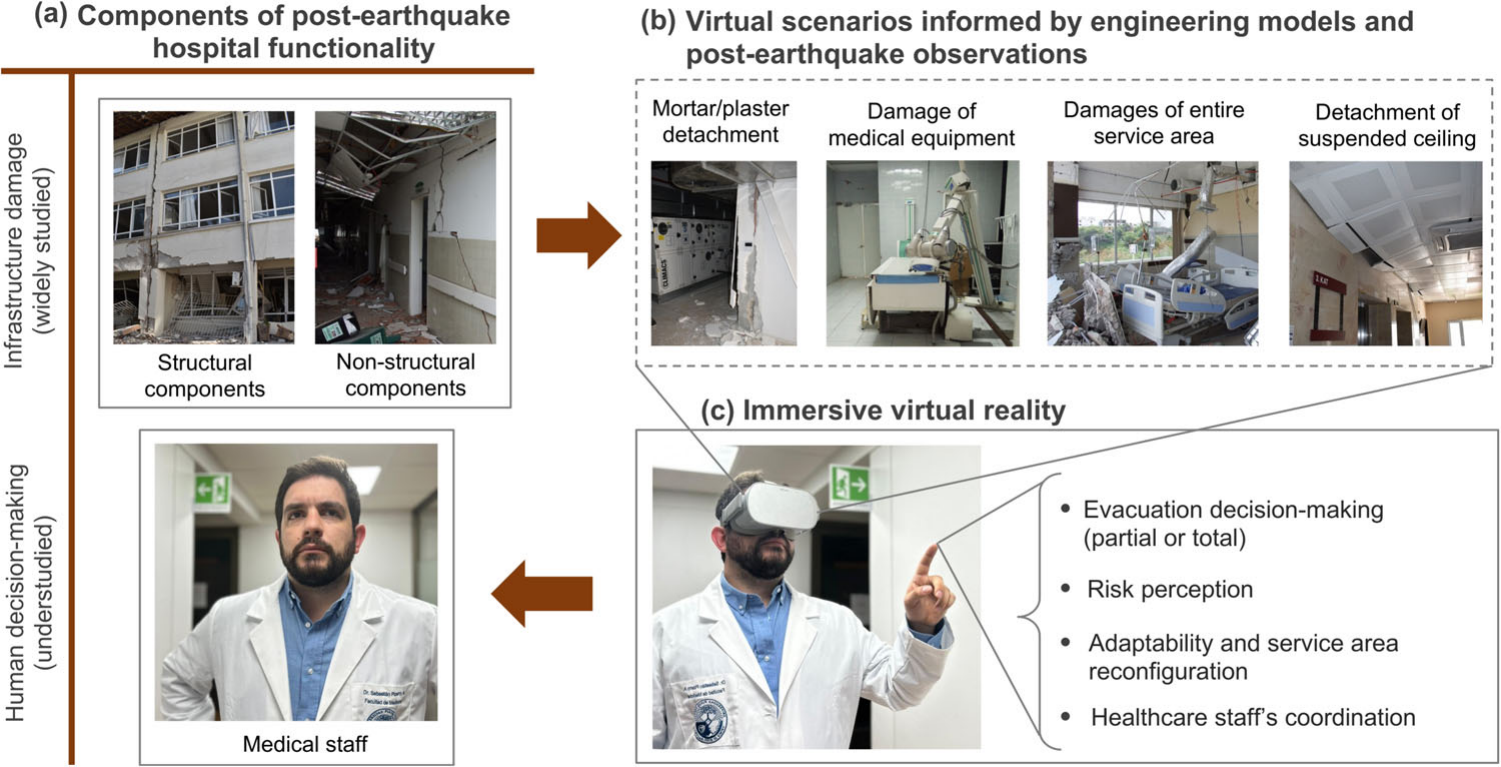
Research opportunities to advance our understanding of post-earthquake hospital functionality. **a** State-of-the-art hospital functionality research focuses on physical infrastructure but lacks the medical staff’s decision-making; (**b**) Engineering models can support the development of realistic, immersive virtual reality (IVR) scenarios of hospital emergencies; (**c**) and these scenarios can help us gather an understanding of the human component of post-earthquake hospital functionality.

Despite the importance of the human component, engineering models that predict post-earthquake hospital functionality largely neglect it. Post-earthquake field investigations primarily focus on physical failures; thus, they cannot collect data about the chief medical staff’s decision-making and untangle their connection with infrastructure damage (Fig. 6a). Here, we propose two exciting avenues to collect behavioral data that can enhance the holistic understanding of hospital vulnerability to earthquakes.

First, field investigations can help us document and reconstruct the functionality of multiple healthcare services and decision-making during emergencies if they are interdisciplinary. Teams composed of engineers, physicians, and social scientists can conduct mixed fieldwork, combining physical damage inspections and semi-structured interviews with medical staff. With this approach, teams can document the functionality of critical services at many timesteps after the earthquake, unveiling their link to limited resources and infrastructure failures. Our reconnaissance team (i.e., this perspective’s authors) followed this approach and reconstructed the functionality of medical services of different hospitals in Türkiye, including the Mustafa Kemal University Hospital. For example, we identified that the power backup system was functional after the earthquake but ran out of fuel in six hours. The hospital could not get fuel and completely lost hospitalization services afterward. This approach also helped us identify that the Mustafa Kemal University Hospital evacuated the main building due to higher perceptions of risk and that the staff reconfigured the hospital services by bringing surgical services to the first floor. While the number of interdisciplinary teams conducting post-disaster reconnaissance is small, encouragingly, such interdisciplinary efforts keep increasing. A few survey tools are already available for interdisciplinary teams to gather this type of data and have already been developed by engineers and public health researchers working across disciplines^93^. However, more deployments are needed to document this information systematically and comprehensively.

Second, immersive virtual reality (IVR) can complement and feed from field investigations to study medical staff’s behavior and decision-making across different post-earthquake scenarios (Fig. 6b). IVR is an effective tool to assess human behavior under multiple simulated scenarios, including earthquakes^95,96^. A recent study has documented healthcare staff’s decision-making (e.g., evacuation) following earthquakes using IVR^97^. While this study has been limited to a unique earthquake scenario and a single medical worker, it hints at the IVR potential to study comprehensive human factors influencing post-earthquake hospital functionality. Future studies using IVR experiments can evaluate, for example, changes in decision-making and risk perception for different damage scenarios to building components, especially if those scenarios are informed by engineering models (Fig. 6c). For example, IVR can be combined with predictions of structural and non-structural building components’ response to seismic ground motions using high-resolution non-linear analysis. This approach opens unique opportunities to study the tipping points in medical staff decision-making, tracking unique behaviors for different damage scenarios, including (i) movement and navigation, (ii) gaze and attention to different hazardous conditions. IVR experiments using multiple (and simultaneous) characters can further give us insights into staff’s collaborative decision-making after earthquakes.

In addition, IVR environments can also be used to better prepare hospital personnel for earthquake emergencies (Fig. 6c). Medical staff can learn to identify possible signs of physical damage to the hospital infrastructure to inform their decisions through IVR, which is key if they need to make rapid decisions and have no structural engineering support, as it often occurs in emergencies. IVR scenarios can also help healthcare staff teams pinpoint potential areas that can support effective and easier reconfigurations, e.g., identifying whether medical staff can find all resources to relocate emergency services to interior or exterior building spaces, as in previous earthquakes.

## Final remarks

This perspective argued that new engineering approaches are needed to enhance seismic safety, opening interdisciplinary research questions and demands for technological solutions. To prioritize areas of relevance, this paper introduced the Earthquake Survival Chain as a framework to identify the most critical infrastructure and services during earthquake emergencies. The chain seeks to augment victims’ survivability and rapidly stabilize communities’ health, especially after large earthquakes^98^.

**Fig. 7:**
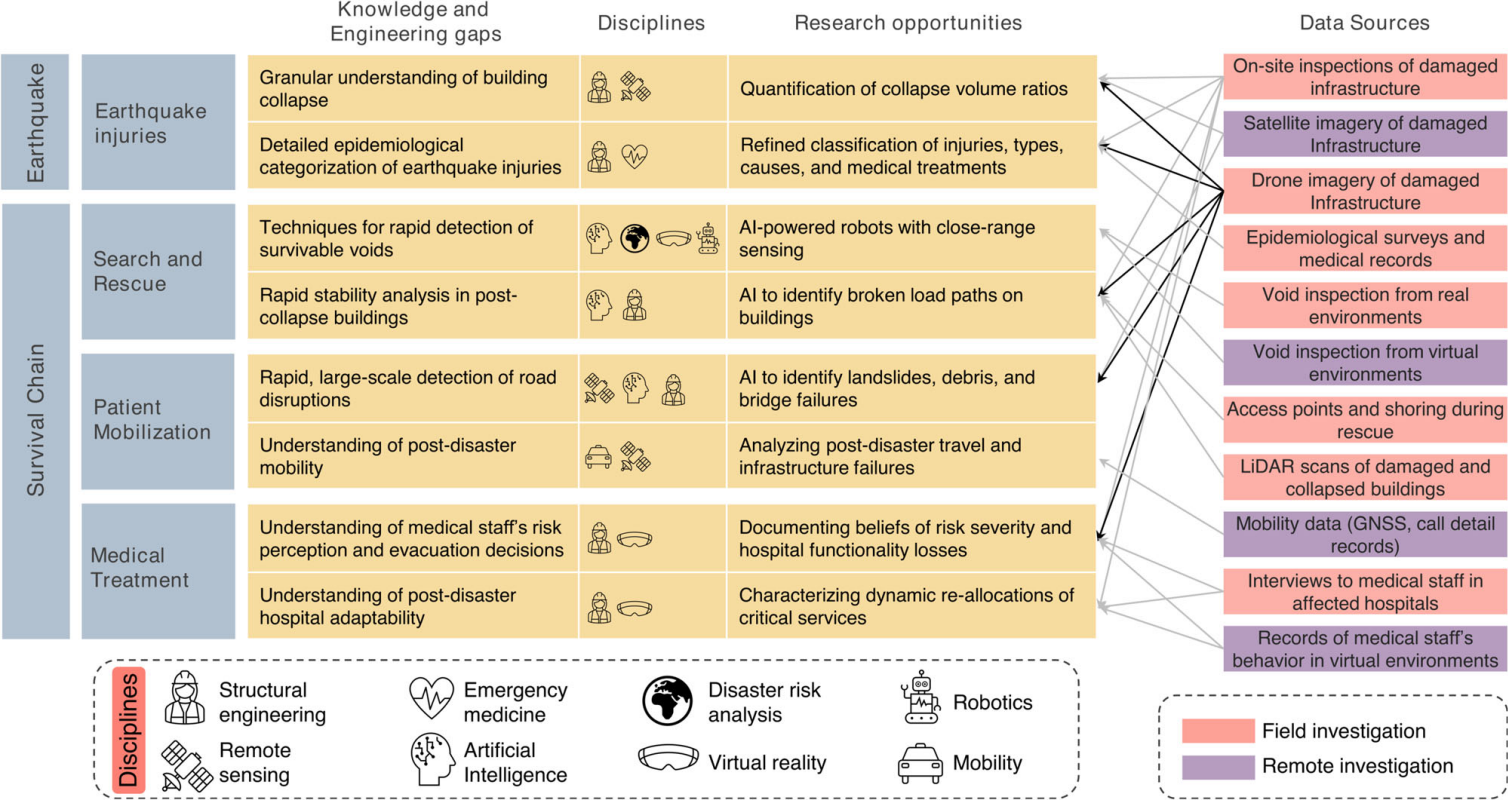
Intersecting multidisciplinary research opportunities and data needs to advance our understanding of and engineer the Earthquake Survival Chain. The arrows highlight how the same datasets can contribute to different research across chain links. In black arrows, we highlight the importance of drone imagery for multiple research directions.

We elaborated on different knowledge gaps and discussed opportunities to improve our understanding of the chain’s links and enhance them. While this perspective described the opportunities for each link separately, they are deeply interconnected, providing encouraging directions to simultaneously strengthen and engineer multiple (or all) components of the earthquake survival chain. Figure 7 summarizes these opportunities for research, including but not limited to the disciplines of structural engineering, remote sensing, emergency medicine, AI, disaster risk analysis, virtual reality, robotics, and mobility. These fields can help enhance multiple links in the earthquake survival chain.

For example, AI can improve search and rescue and patient mobilizations. Robots trained with AI can make searching for trapped people more efficient in large regions. AI can also make the rescue of victims safer by detecting structural instabilities, especially with close-range sensors, in heavily damaged buildings. In addition, AI-powered remote sensing also has tremendous potential for identifying road disruptions, especially when researchers leverage existing AI algorithms for damage detection on buildings.

Immersive virtual reality can also help strengthen more than one chain link. Virtual environments can help us learn and better document USAR teams and medical staff’s decision-making under various synthetic but realistic emergency scenarios, especially if structural engineering models inform the virtual environments.

Similarly, the same datasets can also help enhance multiple links in the chain. For example, comprehensive datasets with granular failure modes of buildings from drones and high-resolution satellite imagery can help improve earthquake injury modeling and, simultaneously, our understanding of stabilities in structures during search and rescue (in highlighted black arrows in Fig. 7). In addition, these datasets can help us better document road disruptions if debris from infrastructure failures blocks streets, hindering patient mobilizations. Further, these datasets can also help us document the hospital damage triggering disruptions of medical treatment.

Promisingly, many datasets that are key for the survival chain do not require field deployments, which are often costly, labor-intensive, and even infeasible sometimes. For example, we can learn from post-disaster satellite imagery remotely and advance earthquake injury modeling, search and rescue, and patient mobilization. As mentioned earlier, virtual reality can also help multiple chain links and be conducted remotely.

Overall, we offer a perspective on data needs and opportunities for interdisciplinary research that can drive the agenda for disaster reconnaissance and earthquake engineering, changing the focus from “infrastructure” to “health and life” to enhance the infrastructure supporting the survival chain in earthquake emergencies.
